# Are loneliness interventions effective in reducing loneliness? A meta-analytic review of 128 studies

**DOI:** 10.1093/eurpub/ckac129.266

**Published:** 2022-10-25

**Authors:** M Lasgaard, C Løvschall, P Qualter, LM Laustsen, MH Lim, HT Maindal, AS Hargaard, J Christensen

**Affiliations:** 1 Public Health and Health Services Research, Defactum, Central Denmark Region, Aarhus, Denmark; 2 Manchester Institute of Education, University of Manchester, Manchester, UK; 3 Department of Clinical Epidemiology, Aarhus University, Aarhus University Hospital, Aarhus, Denmark; 4 Iverson Health Innovation Research Institute, Swinburne University of Technology, Melbourne, Australia; 5 Department of Public Health, Aarhus University, Aarhus, Denmark; 6 Department of Psychology, University of Southern Denmark, Odense, Denmark

## Abstract

Loneliness is widely acknowledged as a growing public health concern, accelerated by the onset of the COVID-19 pandemic. However, our knowledge about the effectiveness of interventions to reduce loneliness across the lifespan, including knowledge of different intervention strategies, is limited. This preregistered systematic review and meta-analysis aimed to evaluate the effect of interventions to reduce loneliness. The systematic review identified 136 studies. The meta-analysis included 128 studies comprising 54 randomised controlled trials (RCTs) (n = 6,379), 23 multi-cohort studies (n = 2,882) and 48 single-cohort studies (n = 3,009). A small to moderate statistically significant effect was detected (RCTs; SMD = -0.47, multi-cohort studies; SMD = -0.24, single cohort-studies; SMD = -0.42). Using the GRADE system, confidence in the estimates was assessed as low or very low, implying that the estimates may potentially be higher or lower. No statistically significant differences were found between age groups. Psychological treatment, social support interventions, and social and emotional skills training appeared to be the most effective intervention strategies in reducing loneliness but there is currently no strong reason to prefer one intervention strategy over another. Further analyses demonstrated that the long-term effects (i.e., one to six months after the intervention) were comparable to the short-term effects (i.e., up to four weeks after the intervention). Findings from the current meta-analyses provide overall evidence of the effectiveness of loneliness interventions. Given methodological limitations, including the heterogeneity of the reviewed studies, it remains unclear who the interventions would help the most. Overall, there is a need for rigorous and high-quality development and evaluations of interventions for loneliness.

**Key messages:**

• The findings of this meta-analytic review suggest that interventions designed to reduce loneliness are effective.

• Psychological treatment, social support interventions, and social and emotional skills training are the most promising interventions, albeit the magnitude of the effects is moderate.

